# Hot water bottle burn injuries: a 10-year retrospective analysis of incidence and potential predictors in Germany

**DOI:** 10.1515/iss-2024-0042

**Published:** 2025-05-22

**Authors:** Moritz Rudolf Milewski, Frederik Schlottmann, Anieto Onochie Matthias Enechukwu, Nico Franke, Martynas Tamulevicius, Louisa Jutta Dietz, Luis Alberto Barros Navarro, Lukas Wellkamp, Khaled Dastagir, Peter Maria Vogt, Vincent März

**Affiliations:** Department of Plastic, Hand, Aesthetic and Reconstructive Surgery, Hannover Medical School, Hannover, Germany

**Keywords:** burns, natural gas, injuries, incidence, hot water bottles, ambient temperature

## Abstract

**Objectives:**

Hot water bottles are widely used for warmth and therapeutic relief, but improper use can lead to burns, ranging from superficial to full-thickness skin injuries. Following the natural gas shortage caused by the complete halt of Russian gas supplies, European countries experienced a sharp increase in hot water bottle-related burns. However, data for Germany were not yet provided. This study examines the incidence of hot water bottle burns over a 10-year period, exploring potential correlations with natural gas prices, natural gas consumption, ambient temperature, and respiratory infection rates.

**Methods:**

A retrospective single-center analysis of 88 patients who sustained hot water bottle burns from 2014 to 2024 was conducted. Patient data, including burn severity and demographic information, were extracted from hospital records. Monthly counts of acute respiratory infections (ARIs), ambient temperature, natural gas price and gas consumption data were also analyzed. A Poisson regression model was applied to assess the association between hot water bottle burns and the mentioned independent variables.

**Results:**

The majority of burns (81.8 %) were second-degree injuries, primarily affecting women (81.8 %). Burns were most common on the lower trunk, thighs, and forearms. The Poisson regression model revealed that for every 1 °C increase in ambient temperature, the incidence of burns decreased by 7 % (IRR=0.93, 95 % CI: 0.88–0.97). However, no significant association was found between ARI incidence, natural gas price and burn occurrence. There was no significant increase of water bottle burns during the recent European energy crisis in Germany.

**Conclusions:**

Hot water bottle burns are more frequent during colder months, particularly among women. Natural gas price or natural gas consumption seems like not playing an equivalent role in Germany as in other European countries. Public health efforts should focus on education and prevention strategies to reduce these preventable injuries. Further research should explore additional factors that may influence burn rates.

## Introduction

Hot water bottles are widely used as a source of warmth and therapeutic relief for a variety of conditions, including muscle pain, menstrual cramps, and the feeling of coldness. However, improper use or defects in these bottles can lead to significant thermal injuries. Burns from hot water bottles, while often considered minor, can range in severity from superficial to full-thickness burns, sometimes necessitating medical intervention or even surgical management [1].

The mechanisms of sustaining burns from hot water bottles vary. Primary causes include spilling hot water during filling, contact heat (even with a Frottee cover), using boiling water, and compromised bottle integrity leading to leakage or rupture [2]. Although easily preventable, hot water bottle burns surged in the first six months of 2023 compared to the previous year, according to the International Burn Injury Database [3]. Specifically, hot water bottle burns increased by 43 % among children and 19 % among adults, becoming a central focus of National Burns Awareness Day in October 2023 [3], 4]. This rise is likely due to the perception of hot water bottles as a cost-effective alternative to central heating, particularly during cold months and periods of high energy prices.

During the 2008 financial crisis, hot water bottle sales and related burns increased in the UK, correlating with high gas and oil prices [2]. Similarly, in 2022, a study by Totty et al. demonstrated a rise in burns caused by various heating devices, including hot water bottles, in the UK during 2022–2023 [5]. The study identified a significant correlation between the European energy crisis – triggered by the complete halt of Russian natural gas supplies – and the increase in burn incidents in the UK. However, while the natural gas shortage and rising prices have been linked to hot water bottle-related burns in the UK, the impact on German households, which rely heavily on natural gas for heating, remains unclear.

Short-term measures by the German government to reduce energy consumption included lowering recommended room temperatures to 19 °C during the cold seasons of 2021/22 and 2022/23 [6]. As a result, natural gas consumption decreased significantly by 18.6 % in 2022 and an additional 5.6 % in 2023 compared to the previous year [7]. While mild weather conditions contributed to this reduction, the lowered room temperatures may have influenced the use of hot water bottles and subsequent burns. On the other hand, the gas price cap alleviated the financial burden on households, potentially reducing the incidence of hot water bottle-related burns.

Despite these measures, data on hot water bottle injuries in Germany remain unavailable. To address this gap, we conducted a retrospective, single-center study over the past 10 years to investigate the factors influencing hot water bottle burns and their trends in Germany. Understanding these predictors is critical for developing targeted prevention strategies and reducing the burden of hot water bottle-related injuries.

## Materials and methods

### Study design and setting

This study was conducted as a retrospective single-center analysis of hot water bottle burn injuries that occurred between 2014 and 2024. Patient data were collected from hospital records. All patients included in the study provided broad consent for the use of their data in research, and as such, no additional patient consent was required for this specific analysis. Ethics committee approval was not necessary, as the use of anonymized patient data falls under the institution’s broad consent policy for retrospective studies.

### Participants

The study population consisted of individuals who sustained burn injuries from the use of hot water bottles during the study period. Inclusion criteria were: (1) confirmed diagnosis of hot water bottle burns, and (2) in- and outpatient treatment at any point between 2014 and 2024. There were no exclusion criteria based on age, gender, or burn severity, and all eligible patients were included in the analysis. The data were collected retrospectively from available medical records.

### Data sources

Five primary datasets were used in this study:

1. Hot Water Bottle Burns Data: Information on hot water bottle burn cases, including the date of injury and patient sex and age were provided by our IT Department and provided via a data warehouse after an extract transform load (ETL) – process of hospital databases. Then, information like total burned surface area (TBSA), burned body part, burn degree and abbreviated burn severity index (ABSI) were extracted from the doctoral letters. Duplicates were excluded. The resulting data frame was thoroughly checked. Minor inconsistencies occurred and were addressed. Using the patient data set, an aggregated burn count table was created for every month in the studied time frame. This count table is published for reuse [8].
2. Acute Respiratory Infections (ARI) Data: Count data on the ambulatory consultation incidence of acute respiratory infections (ARIs) in outpatient clinics in Germany were obtained from Robert Koch Institute (RKI) [9]. These counts summarize infections by rhinovirus, influenza virus, Sars-CoV2 and respiratory syncytial virus (RSV) among others.
3. Temperature Data: A data set of monthly mean temperature data in Hanover, Germany was provided by Deutscher Wetterdienst (DWD), the German National Meteorological Service. Precisely, data from the measuring station 2014 were used [10].
4. Natural gas price: Half annual natural gas price data for German average household consumers were retrieved from Eurostat [11].
5. Natural gas consumption data: Monthly total natural gas consumption data in Germany were retrieved from Eurostat [12].

### Statistical analysis

To assess the relationship between hot water bottle burn injuries, acute respiratory infections, and ambient temperatures, a Poisson generalized linear model (GLM) was applied. The outcome variable was the monthly count of hot water bottle burns, while the predictor variables included the monthly number of acute respiratory infections and the monthly average ambient temperature in Hannover, Lower Saxony, Germany. The natural gas consumption was not included because of the potential non-linear relationship of gas consumption and burns. For example, high gas consumption on mild days typically reflects high indoor temperatures and low burn rates, while low gas consumption on very cold days often indicates underheated homes and higher burn rates. This context-dependent variability makes it difficult to model gas consumption as a direct predictor of hot water bottle-related burns. Additionally, the high variance inflation factor (VIF=8.5) between ambient temperature and gas consumption suggests that including both variables would complicate the interpretation of their individual effects.

To compare cumulative counts of hot water bottle burns during annual cold autumn-winter periods, a Wilcoxon rank sum test with continuity correction was used.

Significance was evaluated at an alpha level of 0.05. Confidence intervals (CIs) were calculated for the model coefficients to provide a measure of precision. The statistical analysis was conducted using R version 4.4.1 for Mac (R Foundation for Statistical Computing, Vienna, Austria) [13].

### Plotting

Ggplot2 [14] package was used to produce the combined line, dot and bar plots. The package humapr [15] was used to create the chloropeths of the human body.

### Reporting

All results were reported in accordance with the STROBE guidelines for observational studies [16].

### Bias

Given the retrospective nature of the study, there is a selection bias due to the reliance on available hospital records. Additionally, children are completely missing in our data set.

### Study size

The sample size was determined retrospectively, based on the number of patients presenting with hot water bottle burns between 2014 and 2024. The study was not designed with a predetermined sample size or power calculation.

## Results

Eighty-nine patient records were obtained as provided by our internal IT service. One duplicate had to be removed. After examining every patient, everyone of the 88 remaining patients could be included into our study due to sustained hot water bottle injuries.

### Demographics

**Figure 1:**
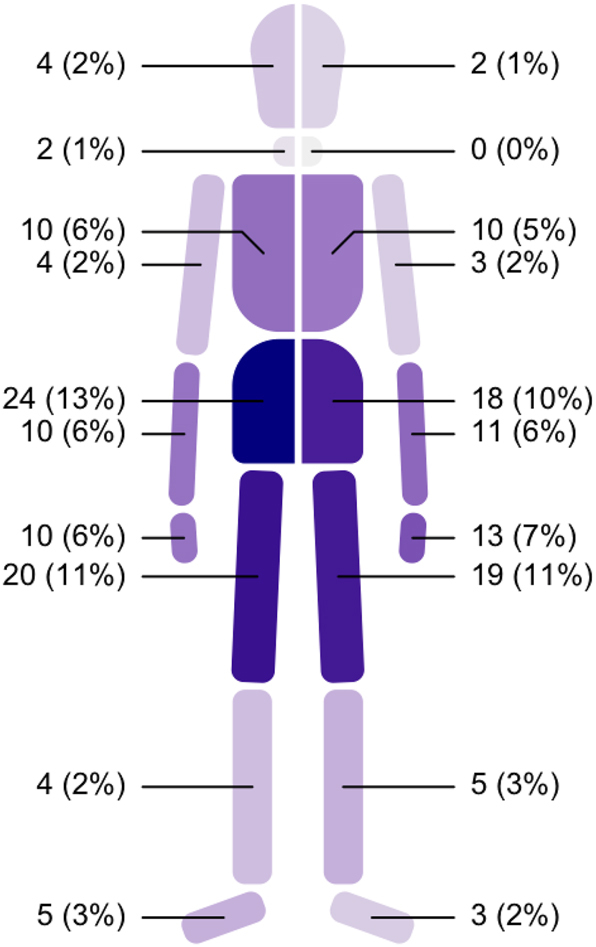
Distribution of injured body parts. The figure illustrates the distribution of burns caused by hot water bottles across different body parts, presented as a human plot. Each body section is color-coded from light to dark blue to indicate the frequency of burns, with darker shades representing higher frequencies. Absolute numbers of burns are displayed for each body part, followed by the corresponding frequency in parentheses. The color gradient reflects the frequency of burns and does not indicate the thickness or severity of the injuries. The most frequently affected areas include the lower abdomen and thighs.

Patient demographics are detailed in Tables 1 and 2. Our data indicate a significantly higher incidence of hot water bottle injuries among women during the study period. The majority of burns were located on the lower trunk, thighs, and forearms (Figure 1). Burn severity was predominantly classified as second-degree, superficial partial-thickness (2a), with a statistically significant distribution pattern (p<0.0001). A total of 23 patients required admission to our specialized burn treatment unit (hydrotherapy), where they received thorough wound cleaning, blister debridement, and appropriate dressings. Wound care involved either polyhexanide gel (LAVANID^®^, SERAG-WIESSNER GmbH & Co. KG, Naila, Germany) with fatty gauze, or Suprathel^®^ (PolyMedics Innovations GmbH, Kirchheim unter Teck, Germany). Only four patients needed split thickness skin grafting. The total body surface area (TBSA) affected by burns was relatively low, with a mean (M) of 3 % and a standard deviation (SD) of 3.1 %.

**Table 1:** Patient demographics table of discrete variables.

Category	Absolute count	Frequency, %	p-Value	Χ^2^	df
Gender					
Female	72	81.8 %	p<0.0001	35.36	1
Male	16	18.2 %			
Body parts					
Forearm	17	19.3 %	p<0.0001	85.38	9
Arm	6	6.8 %			
Thigh	32	36.4 %			
Leg	6	6.8 %			
Lower trunk	36	40.9 %			
Hand	20	22.7 %			
Foot	8	9.1 %			
Upper trunk	17	19.3 %			
Head	4	4.5 %			
Neck	2	2.3 %			
Burn degree					
First-degree	25	28.4 %	p<0.0001	93.8	3
Superficial partial-thickness	72	81.8 %			
Deep partial-thickness	13	14.8 %			
Third-degree	5	5.7 %			
Treatment					
Hydrotherapy	23	26.1 %	p<0.0001	73.2	3
Split thickness skin grafting	4	4.5 %			
Suprathel®	18	20.5 %			
Polyhexanide gel	64	72.7 %			

**Table 2:** Patient demographics table of continuous variables.

Variable	n	Mean	SD	Min	Percentile 25	Percentile 75	Max
Age	88	38	18	11	25	50	86
TBSA in percent	88	3	3.1	0.1	1	4	18
ABSI	7	4	0.82	3	3.5	4.5	5

### Exemplary case with follow-up

Due to post-treatment burn care primarily occurring in external facilities, only one patient with severe burns (exceeding 5 % TBSA) returned for follow-up five months post-injury. This patient, a middle-aged woman, had sustained second-degree burns on the right gluteal region and the dorsal aspect of the right thigh after a hot water bottle burst. Initial evaluation and debridement were performed at another hospital two days after the incident. Subsequently, the patient was transferred to our facility for further treatment due to signs of infection.

**Figure 2:**
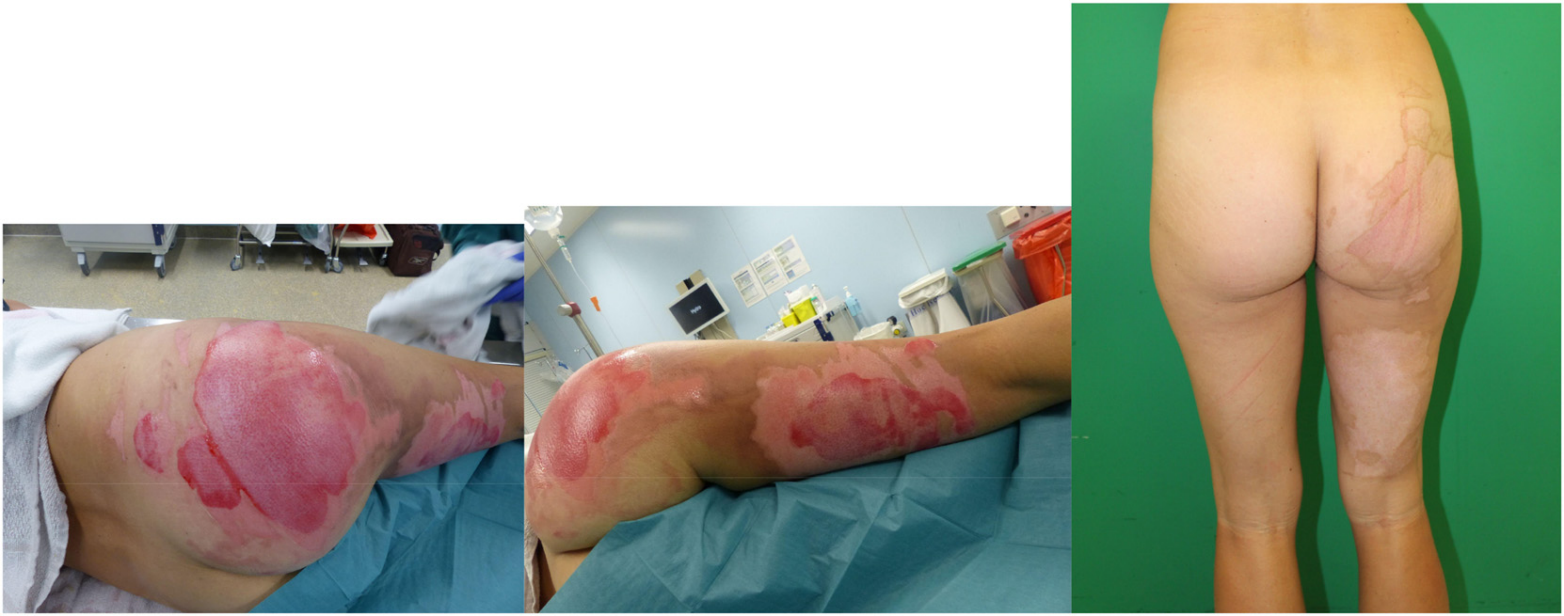
Wounds after initial debridement and after five months (from left to right).

Upon admission, a conservative wound management approach was implemented. Polyhexanide gel, combined with fatty gauze, was applied to the affected areas to manage the infection. The extent of the burns and the presence of infection necessitated close monitoring to determine if surgical intervention might be required. However, the wound showed a positive healing response, and surgery was ultimately unnecessary. At five months post-injury, the aesthetic and functional outcomes were favorable (Figure 2).

### Outcome data

**Figure 3:**
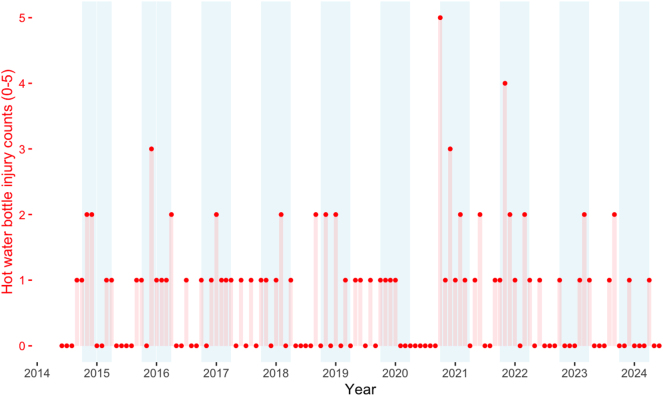
Monthly distribution of hot water bottle injuries (2014–2024). This Figure illustrates the monthly count of hot water bottle injuries over a 10-year period, from 2014 to 2024. Red dots and bars represent the number of injuries per month, with the exact counts displayed on the left y-axis. The x-axis shows each year, providing a clear timeline of injury trends. Light blue vertical bars highlight the cold months (October–March), during which the use of hot water bottles is more prevalent.

**Figure 4:**
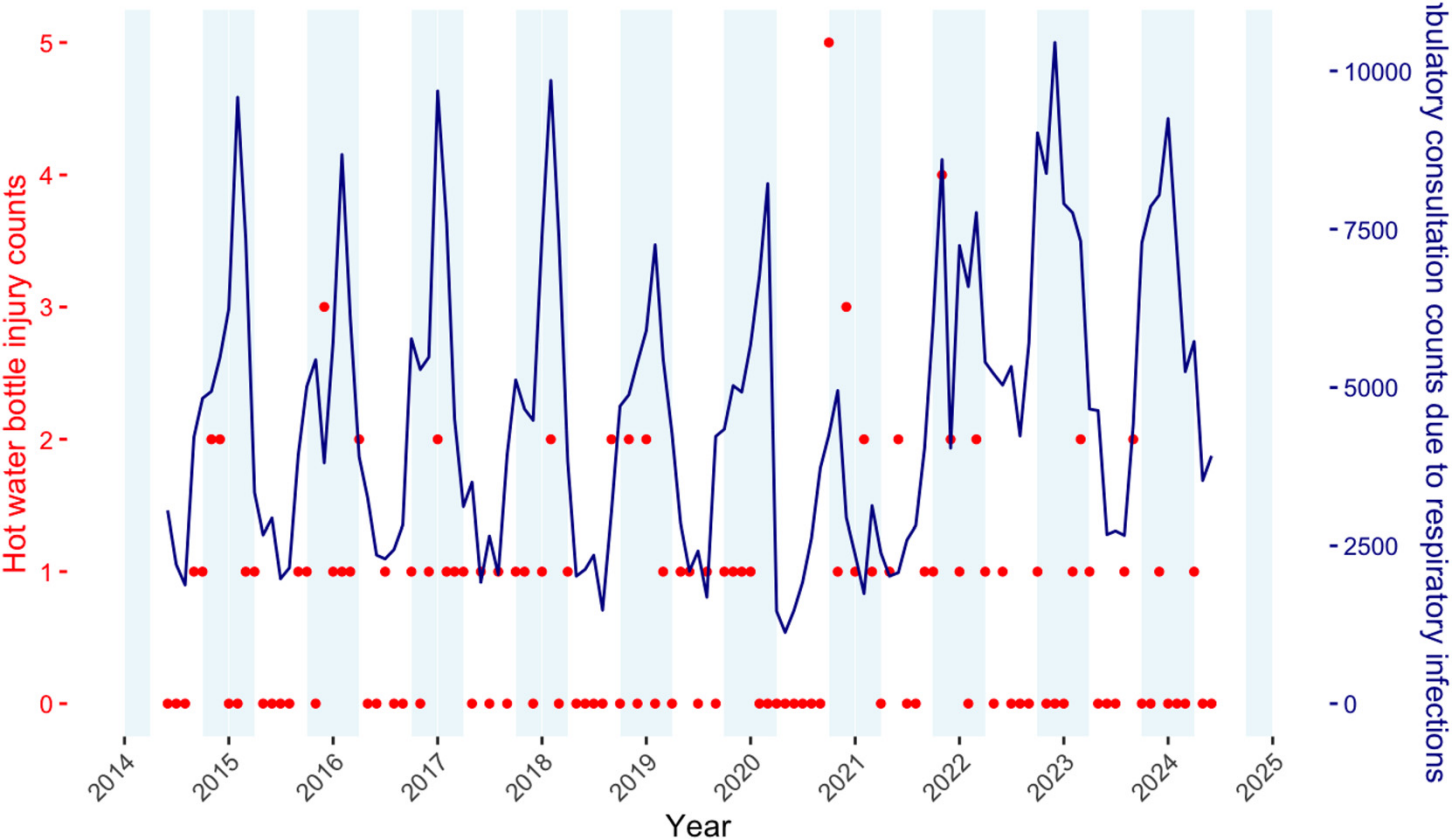
Hot water bottle injuries and respiratory infections (2014–2024). The dark blue line shows monthly counts of ambulatory patients with respiratory infections (e.g., rhinovirus, influenza, severe acute respiratory syndrome coronavirus 2 [SARS-CoV-2], or respiratory syncytial virus [RSV]). Dots represent hot water bottle injuries. The figure explores whether infection peaks correlate with increased hot water bottle use and injury rates. Please compare with Figure 3.

**Figure 5:**
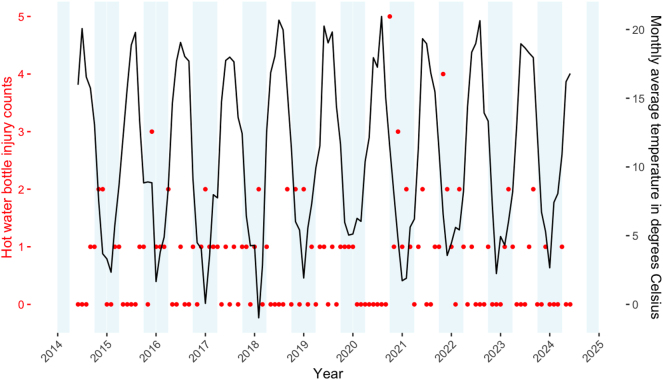
Hot water bottle injuries and monthly mean temperature in Hannover, Germany (2014–2024). The black line shows the monthly mean temperature in Hanover, Germany, in degrees Celsius. Dots represent hot water bottle injuries. The figure explores the relationship between temperature and injury rates, with colder months potentially correlating with higher burn incidence. Please compare with Figure 3.

**Figure 6:**
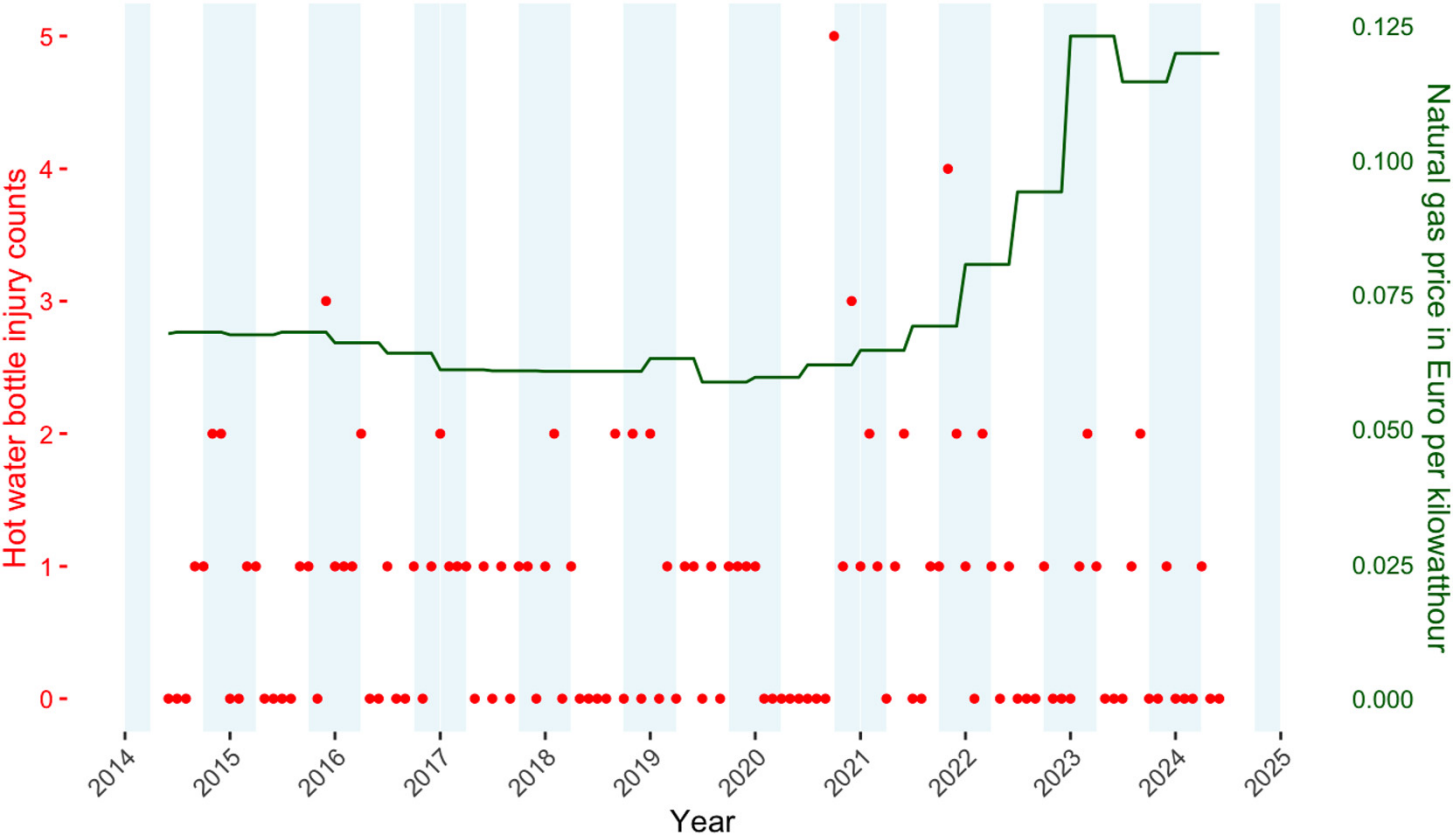
Hot water bottle injuries and natural gas prices for German households (2014–2024). The green line shows the half-annual natural gas price in euros per kilowatt-hour paid by the average German household. Dots represent hot water bottle injuries. The figure explores the relationship between gas prices and injury rates, with higher prices occurring after 2022. Please compare with Figure 3.

**Figure 7:**
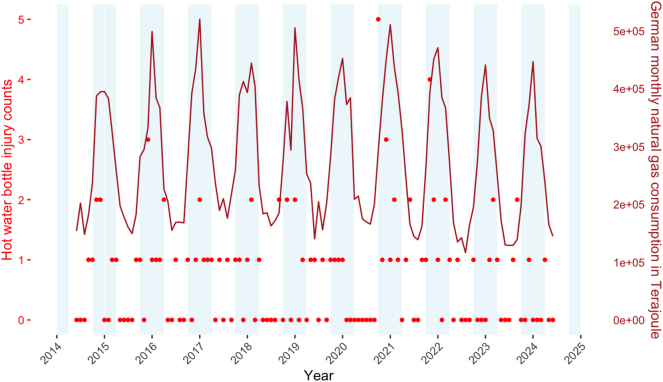
Hot water bottle injuries and natural gas consumption in Germany (2014–2024). The brown line shows the total German natural gas consumption in terajoules in one month. Dots represent hot water bottle injuries. Please compare with Figure 3.

As mentioned, 88 patients treated in our department between 2014 and 2024 sustained hot water bottle-related injuries. A histogram of monthly aggregated counts is provided in Figure 3, showing that hot water bottle injuries were generally one or fewer per month, with a few notable exceptions, particularly during late 2020 and early 2021. Interestingly, after the rise of COVID-19 in 2020, overall acute respiratory infection (ARI) rates were comparatively low, and no hot water bottle-related admissions were recorded for eight consecutive months (Figure 4). When comparing the incidence of ARIs in outpatient clinics to hot water bottle burns, a notable overlap is observed between ARI peaks and the colder months from October to March (Figures 4 and 5). As expected, the mean ambient temperature in Hanover was significantly lower from October to March compared to the rest of the year, with the mildest mean temperatures recorded during the autumn-winter period of 2019/2020 (Figure 6). Concurrently, the price of natural gas in Germany rose sharply from 2021 to 2023 (Figure 6). Gas consumption closely mirrored temperature trends, with higher consumption during colder months and lower consumption during warmer periods (Figure 7).

### Poisson model

Our first aim was to find predictors of water bottle occurrences. Thus, we used a Poisson regression model to assess the association between the monthly count of hot water bottle burn injuries and three predictors: The monthly incidence of acute respiratory event reported by outpatient clinics in Germany, the ambient temperature in Hanover, Germany, and the kilowatt hour price in EUR for natural gas consumed by a typical German household. The model’s log-link function is appropriate for count data and ensures non-negative predictions.

The model’s goodness-of-fit statistics were as follows: null deviance: 129.16 on 114 degrees of freedom; Residual deviance: 114.96 on 111 degrees of freedom; AIC: 258.28; Chi-squared test for goodness of fit: p=0.37. These values suggest that the model provides a reasonable fit to the data. The deviance residuals are not excessively large, indicating no major issues with the model’s fit. The number of Fisher scoring iterations was 5, confirming convergence. Thus, ambient temperature was a significant predictor (p=0.003) (Table 3). The Incidence Rate Ratio (IRR) of 0.93 indicates that for every 1 °C increase in temperature, the rate of hot water bottle burn events decreases by 7 %, holding other factors constant (IRR=0.93, 95 % CI: 0.88–0.97). The monthly incidence of respiratory infections did not have a statistically significant effect on the rate of burns (p=0.63), with a negligible decrease in the event rate per unit increase in ARI incidence (IRR=1.00, 95 % CI: 1.00–1.00). Gas price also did not significantly affect the rate of hot water bottle burns (p=0.63), and its IRR (0.02) suggests that variations in gas price do not have a meaningful or consistent impact on injury counts (95 % CI: 0.00–398.59).

**Table 3:** Poisson model results.

Predictor	Coefficient (β)	Standard error	Z-value	p-Value	Incidence rate ratio (IRR)	95 % CI of IRR
Intercept	0.78	0.50	1.56	0.12	2.18	0.84–5.64
Monthly ARI incidence	0.00	0.00	−0.48	0.63	1.00	1.00–1.00
Temperature	−0.08	0.03	−2.96	0.003	0.93	0.88–0.97
Gas price	−4.01	8.30	−0.48	0.63	0.02	0.00–398.59

**Figure 8:**
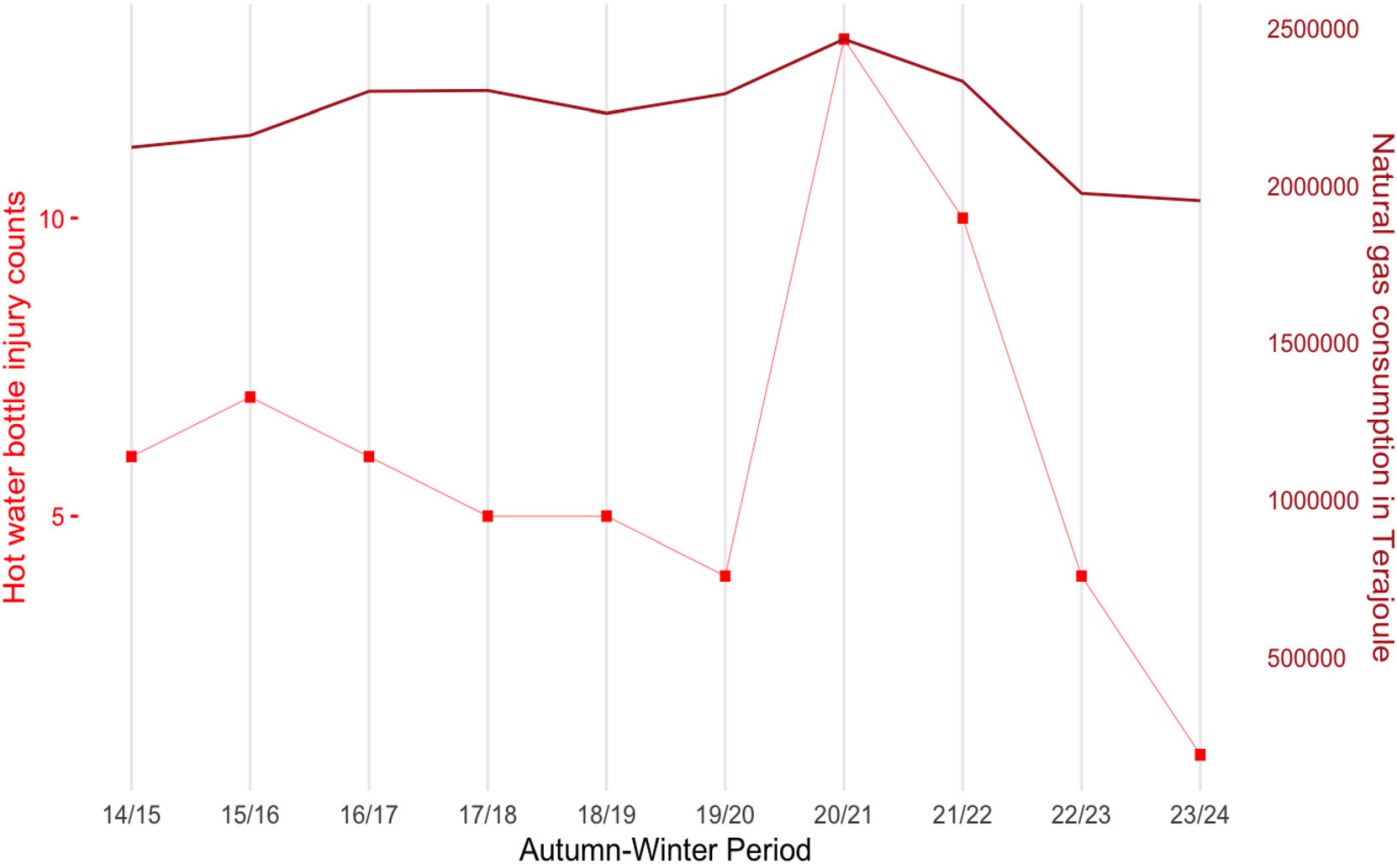
Comparison of hot water bottle injury counts and cumulative natural gas consumption for each cold period (2014–2024). Burn counts and natural gas consumption were pooled for each cold period (October–March). Burn counts are depicted as connected red dots, while cumulative natural gas consumption is shown as a brown line. Total hot water bottle burns are scaled on the left y-axis (0–15), and cumulative natural gas consumption is scaled on the right y-axis in terajoules. The figure explores the relationship between gas consumption and injury rates during colder months, with no visual correlation between the two. Please compare with Figure 7.

The second aim was to investigate differences in the occurrence of burn injuries considering the autumn-winter period from 2022/23 and 2023/24 and compare them to the other cold periods without natural gas shortage (Figure 8). Comparing the burn counts, in 23/24 only one patient presented with hot water bottle burns, being drastically lower than in other years. A Wilcoxon rank sum test with continuity correction was conducted to compare the burn counts in the cold period 2023/24 to those in years without gas shortage (2021/22 and before) but we failed to reject the null hypothesis that the true location shift is equal to 0 (W=8, p=0.17). In other words, there was no statistically significant difference between the burn counts in 2023 and the cold periods before.

## Discussion

We conducted a retrospective, single-center study over a 10-year period to assess the incidence of hot water bottle burns during the European energy crisis that followed the onset of the Russian–Ukrainian war. The demographic data were consistent with prior studies, revealing that women predominantly suffered second-degree (2a)/superficial partial-thickness burns [17].

One reason why more women experience burns from hot water bottles may be the longstanding use of heat as a non-pharmacological remedy for period cramps. Heat helps relax the smooth muscle fibers of the uterus, providing relief [18]. This connection is also reflected in the comparatively young, possibly still reproductive, age of many patients affected by these burns. At the end of 2021, the average age in Hanover was 42.61 years (men: 41.24 years, women: 43.95 years), which is considerably older than the patient population mean age of 38 years ± 18 years [19]. The low TBSA is consistent with findings from a recent meta-analysis, which also reported relatively low burn surface areas across hot water bottle burns [20]. However, the frequently burned lower abdomen, including the perineal area, and the thigh region are particularly vulnerable to complications in burn cases due to frequent exposure to fecal and urinary contamination, which presents added challenges for wound care providers [21].

It is worth noting that treatment for scald burns is typically conservative, meaning that surgery is often not required. In our study, only 4.5 % of burns required surgical intervention, even though 5.7 % of the burns were classified as third-degree. This discrepancy suggests that either the burns were small enough to be managed conservatively, or patients opted out of surgical treatment, despite third-degree burns generally being an indication for surgery.

Our findings indicated a decline in hot water bottle burns in 2022 and 2023 compared to previous years, despite high gas prices and reduced gas consumption. This decrease coincided with relatively mild winter temperatures. In contrast, a UK study observed a significant increase in burns during the winter of 2022/2023, with many linked to heating devices, including hot water bottles [5].

Our Poisson regression model of hot water bottle burn counts revealed that only ambient temperature had a significant correlation, whereas the number of respiratory infections and gas prices did not. This suggests that hot water bottles may be used primarily for comfort when feeling cold, rather than to alleviate symptoms of illness. Additionally, it appears that Germans may have been more hesitant to rely on hot water bottles as an alternative to heating their homes during the gas price shocks of 2022 and 2023. This hesitancy might correlate with differences in socioeconomic status between Germany and the UK, which could have influenced hot water bottle burns eventually. In the UK, it is common practice to use prepayment meters, which require frequent charging to access water, electricity, and gas [22]. These meters are particularly prevalent among low-income households. As a result, it is not surprising that two million UK households were at risk of going without heating and electricity in 2022 [23]. However, it is important to note that correlation does not imply causality, and further research is needed to establish direct causes. Multicenter, prospective studies could provide more clarity on the factors contributing to hot water bottle burns.

This study has several limitations. First, the findings are based on data from a single burn center in Hannover, Germany, which may limit generalizability to populations in other regions with differing climates, energy policies, cultural practices, or healthcare access. Second, retrospective data collection introduces potential biases. Reliance on hospital records may lead to underreporting of mild burns treated in outpatient settings or undocumented cases. Crucially, the retrospective identification of cases through keyword searches in doctoral letters risks missing cases with non-standard terms or omitted causative details. Third, pediatric cases were not presented to our hospital, as such patients are typically referred to specialized pediatric centers. This prevents insights into hot water bottle burns among children – an extraordinarily vulnerable population. Additionally, we are unable to provide a detailed breakdown of injury etiologies due to the retrospective nature of the study and the lack of standardized reporting on causation. Finally, the small sample size (88 cases over 10 years) reduces statistical power to detect subtle associations. Retrospective designs also preclude causal inferences, as unmeasured confounders (e.g., household income, educational status, or behavioral factors) could influence both hot water bottle use and injury risk.

Despite these limitations, the study provides valuable insight into a preventable cause of thermal injury. Public health campaigns should focus on educating people about the safe use of hot water bottles, such as the importance of avoiding boiling water and regularly inspecting bottles for wear and tear. Manufacturers could improve safety by enhancing the durability of hot water bottles and incorporating features like temperature indicators to prevent overheating. Regulatory bodies could also introduce stricter safety standards for the production and sale of hot water bottles to minimize the risk of defects that may cause burns. Eventually, the usage of alternatives to hot water bottles, such as cherry stone or infrared pillows, may reduce the risk of scalds from water spillage or malfunction. However, they still carry a risk of contact burns. The efficacy of these endeavors could be proven in Australia, where strict legislation and media campaigns in the 2000s led to a decrease in hot water bottle burns from 10 burns in a single center in 2004/05 to two burns in 2008/09 [24].

In conclusion, while hot water bottle burns may not represent the majority of thermal injuries, their preventable nature and potential for increased incidence during periods of cold weather justify focused prevention efforts. By addressing this issue, we can reduce the burden of these injuries and improve outcomes for affected individuals.
